# Trait-Like Brain Activity during Adolescence Predicts Anxious Temperament in Primates

**DOI:** 10.1371/journal.pone.0002570

**Published:** 2008-07-02

**Authors:** Andrew S. Fox, Steven E. Shelton, Terrence R. Oakes, Richard J. Davidson, Ned H. Kalin

**Affiliations:** 1 Department of Psychology, University of Wisconsin-Madison, Madison, Wisconsin, United States of America; 2 Department of Psychiatry, University of Wisconsin-Madison, Madison, Wisconsin, United States of America; 3 The Waisman Laboratory for Brain Imaging and Behavior, University of Wisconsin-Madison, Madison, Wisconsin, United States of America; University of Parma, Italy

## Abstract

Early theorists (Freud and Darwin) speculated that extremely shy children, or those with anxious temperament, were likely to have anxiety problems as adults. More recent studies demonstrate that these children have heightened responses to potentially threatening situations reacting with intense defensive responses that are characterized by behavioral inhibition (BI) (inhibited motor behavior and decreased vocalizations) and physiological arousal. Confirming the earlier impressions, data now demonstrate that children with this disposition are at increased risk to develop anxiety, depression, and comorbid substance abuse. Additional key features of anxious temperament are that it appears at a young age, it is a stable characteristic of individuals, and even in non-threatening environments it is associated with increased psychic anxiety and somatic tension. To understand the neural underpinnings of anxious temperament, we performed imaging studies with 18-fluoro-deoxyglucose (FDG) high-resolution Positron Emission Tomography (PET) in young rhesus monkeys. Rhesus monkeys were used because they provide a well validated model of anxious temperament for studies that cannot be performed in human children. Imaging the same animal in stressful and secure contexts, we examined the relation between regional metabolic brain activity and a trait-like measure of anxious temperament that encompasses measures of BI and pituitary-adrenal reactivity. Regardless of context, results demonstrated a trait-like pattern of brain activity (amygdala, bed nucleus of stria terminalis, hippocampus, and periaqueductal gray) that is predictive of individual phenotypic differences. Importantly, individuals with extreme anxious temperament also displayed increased activity of this circuit when assessed in the security of their home environment. These findings suggest that increased activity of this circuit early in life mediates the childhood temperamental risk to develop anxiety and depression. In addition, the findings provide an explanation for why individuals with anxious temperament have difficulty relaxing in environments that others perceive as non-stressful.

## Introduction

Early in life, the expression of extreme BI, characterized by excessively inhibited responses to strangers or novel situations is a considerable risk-factor for the development of anxiety and depression [1]–[3]. Additionally, some children with extreme BI have increased pituitary-adrenal and autonomic activity suggesting that these individuals have a dispositional tendency to activate stress-related systems [3]. Youth with this disposition are considered to have an “anxious temperament”, in part because they have excessive activation of behaviors and physiological responses that are associated with stress and anxiety and also because they are more likely to develop anxiety disorders and depression later in life. Since anxious temperament can be identified early in childhood and it is a risk factor for the development of anxiety and depression, it is important to understand the mechanisms in the developing brain that underlie its expression. An important feature of anxious temperament is that it is a stable behavioral and emotional style that is apparent across different environments. For example, children with anxious temperament are extremely inhibited when confronted by unfamiliar individuals and in less threatening situations appear to have increased levels of anxiety, worry, and physical tension. While it is normal to show increased levels of anxiety in highly novel and unfamiliar situations, it is maladaptive to express anxiety in a highly familiar context [4]. Therefore, to understand the mechanisms underlying anxious temperament it is critical to identify the relations between brain function and anxious temperament that are stable over time and invariant across both stressful and secure environments. Such studies are not feasible in children but can be performed in a well established rhesus monkey model of anxious temperament.

**Figure 1:**
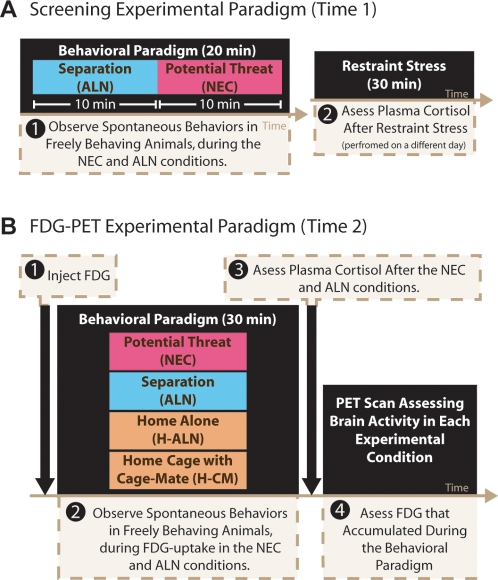
Animals were tested two times: screening (a) and FDG-PET (b). Behavioral and physiological screening were performed on separate days. During each behavioral screening animals were left alone in the test cage (ALN, blue) for 10-minutes, followed by a 10-minute exposure to a human intruder presenting her profile to the monkey while making No Eye Contact (NEC, pink) (a-1). To obtain a physiological measure of stress, plasma cortisol was collected after exposure to 30 minutes of restraint stress (a-2). Approximately 4 months later, animals were assessed on separate days with four FDG-PET scans, two in stressful conditions (ALN & NEC) and two conditions in the security of their home-cage (seen in orange); one condition in which they were alone (H-ALN) and one condition where their cage-mate was present (H-CM). On each test day, animals were injected with FDG (b-1) and exposed to 30-minutes of the behavioral paradigm. During stressful conditions, behavior was monitored (b-2) and plasma cortisol was collected after the cessation of the stress (b-3). FDG-uptake associated with in condition brain metabolism was assessed after completion of the behavioral paradigm, (b-4).

In a prospective longitudinal study, we assessed neural activity and its relation to the behavioral and physiological markers of anxious temperament in young rhesus monkeys that varied in their degree of trait-like anxiety. To achieve this, the monkeys were studied in different stressful environments involving relocation and exposure to a potential-threat, as well as in the security of their home cage. Young rhesus monkeys are ideal for this work because numerous studies validate their use in modeling childhood anxious temperament [5]. The different stressful contexts were selected because they evoke different types of coping responses that are in part modulated by different neurochemical systems [6]. Because of the importance of assessing brain function that occurs in naturalistic and relevant settings, we administered 18-fluoro-deoxyglucose (FDG) to the monkeys in the environment of interest and later, with high-resolution Positron Emission Tomography (PET), imaged the regional uptake of FDG that occurred during that period (Fig. 1). We predicted that early in life, the amygdala, a region with the capacity to activate multiple systems underlying the adaptive behavioral and physiological responses to stress, would be associated with the behavioral and physiological components of anxious temperament. Such a finding would be consistent with an fMRI study demonstrating increased amygdala reactivity in human adults who at 2 years of age were characterized as behaviorally inhibited [7]. In addition research in non-human primates demonstrates that selective amygdala lesions alter stress and anxiety-related behavior and physiological responses [8], [9]. We further hypothesized that this relation between temperament and brain metabolic activity would be evident across different contexts that varied in their type and degree of stressfulness, including the subjects' home environment. To assess anxious temperament we used a composite measure that reflects the activation of stress-related behavioral and physiological responses. In contrast to using a single behavioral or hormonal measure, a composite measure of behavior and physiology more broadly reflects individual differences across the multiple systems that are involved in the stress response.

## Results

To ensure variability in the anxious temperament of the animals that were used, we screened 116 periadolescent monkeys selecting 36 animals with the most stable high (n = 12, 9 female and 3 male), middle (n = 12, 8 female and 4 male) and low (n = 12, 9 female and 3 male) levels of threat-induced BI or freezing behavior (average age+SEM at screening = 2.3+.09 yrs.; range = 1.5–3.4 yrs; puberty onset in rhesus monkeys is approximately 3 yrs). Although more females than males were selected for further study, the overall stability in freezing behavior, as measured by the change in the amount of freezing between the two screening days, did not significantly differ between males and females (t(114) = −1.601, p = .112). For initial selection and categorization, freezing was assessed by separating the monkey from its cage mate and relocating it to a test cage in which for 10 minutes it was exposed to the potential threat of a human intruder presenting her facial profile to the monkey [no eye contact condition (NEC) of the human intruder paradigm] (Fig. 1a). Although freezing in response to NEC is considered adaptive, excessive freezing is analogous to extreme childhood BI [5], [6]. In addition to measuring freezing behavior, individual differences in spontaneous coo vocalizations were assessed for 10 minutes immediately prior to the NEC condition while separating the monkey from its cage mate and relocating it to the test cage [alone condition (ALN) of the human intruder paradigm] (Fig. 1a). The ALN condition typically elicits separation induced coo vocalizations, which can be likened to calls for help. Less coo calling is associated with increased anxiety and increased amygdala activity [10]. In addition to the behavioral measurements, each animal had blood drawn on two separate occasions following exposure to restraint stress, to assess stress-induced plasma cortisol levels.

On an average of 4.3 months later, brain activity was measured on 2 separate occasions with FDG-PET in 35 of the 36 monkeys during 30-minutes of exposure to the NEC and ALN conditions (one monkey that displayed high levels of freezing during screening was not scanned because she refused to leave her cage). The 30-minute period of stress exposure was selected because FDG enters active brain cells during a 30-minute uptake period, and remains stably detectable within these regions for prolonged periods (due to its 110-minute half-life) [11], [12]. This time course also allows for later anesthesia administration and imaging of the brain activity that occurred during the preceding period of stress exposure (Fig. 1b). During the period of FDG uptake, NEC-induced freezing and ALN-induced cooing were assessed as increased BI, and decreased spontaneous vocalizations that are characteristic features of children with anxious temperament. Blood was collected at the end of these conditions to assess stress-induced cortisol levels. To create a composite measure of anxious temperament, we first z-scored each measure of stress responsivity (increased freezing during NEC, increased cortisol levels in response to NEC, decreased cooing during ALN, and increased cortisol levels in response to ALN; controlling for any age effects across all animals) across subjects and then computed the mean of the four z-scored measures for each subject. To understand the extent of the relation between the individual measures comprising the composite score, correlations between the individual variables (after being age-residualized and z-scored) were performed. Freezing during NEC and cooing during ALN were marginally negatively associated (r(33) = −.332, p = .052); all other pairs of variables were not significantly correlated (r's(33)<.253, p's>.143). Because the distribution of the composite measurement of anxious temperament did not differ from a normal distribution (Kolmogorov-Smirnov one-sample test against a normal distribution (KS)(n = 35) = .602, p = .861) and revealed no discernable subgroups, we treated anxious temperament as a continuous measure in all subsequent analyses.

**Figure 2:**
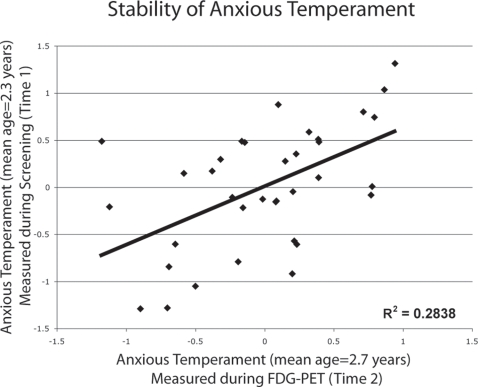
Anxious temperament, defined as increased freezing during NEC, decreased cooing during ALN and increased stress-induced cortisol, assessed during screening was significantly correlated with a similar measure taken 4-months later during FDG-PET testing.

Since anxious temperament in children is relatively stable, we examined the extent to which this composite measure of stress responsivity was correlated with the 35 monkeys' composite measure of stress-related behavior and physiology that was collected 4.3 months earlier during their initial assessment [3], [13]. Consistent with the trait-like qualities of anxious temperament, results demonstrated that this composite measure was stable (r(33) = .533, p<.001) (Fig. 2). In a subsequent study involving 24 of these animals, the same parameters were assessed at 3.8 years of age or 1.5 years later. Results revealed that individual differences in stress responsivity remained stable as the animals matured (r(22) = .460, p = .027). We observed no gender differences (t's(33)<1.045, p>.304) or evidence of non-normal distributions (KS's(n = 35)<.923, p's>.361) within any of the composite measures of anxious temperament. These data demonstrate that a composite assessment of stress-related behavioral and physiological measurements reflecting anxious temperament is stable over development in non-human primates.

**Figure 3:**
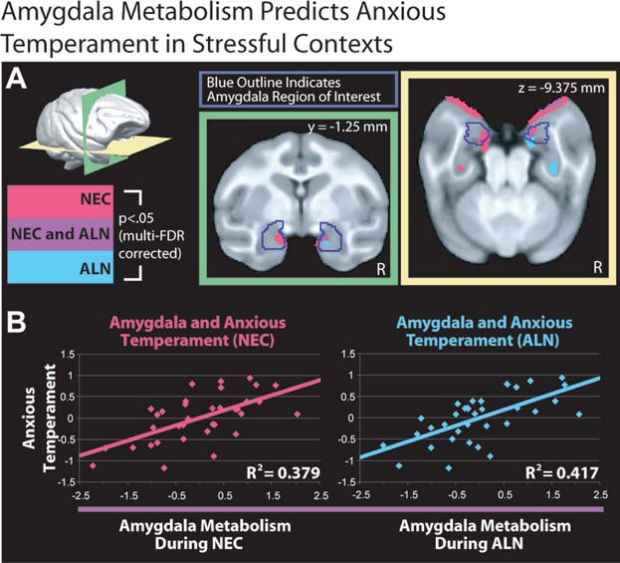
a) Significant (p<.05, two-tailed multi-FDR corrected) relationships between regions of the amygdala (outlined in blue) and anxious temperament (Time 2) during the ALN (cyan) and NEC (pink) conditions are overlaid on coronal (green-border) and axial (yellow-border) slices of the study-specific MRI template (see Supplementary Methods). The areas of the brain demonstrating significant correlations in both the NEC and ALN conditions are shown in purple. b) Scatter-plots show anxious temperament (Time 2) to be correlated with z-scored residual metabolism in this overlapping region of the amygdala (after accounting for age and gray-matter probability) in both the NEC (pink, left) and ALN (cyan, right) conditions. Coordinates are in millimeters relative to the anterior commisure.

To assess the relation between individual differences in anxious temperament and brain metabolism, we performed, separately for the NEC and ALN conditions, voxelwise correlations between monkeys' anxious temperament scores and brain metabolism, while controlling for age. To account for anatomical differences and any small errors in inter-subject registration that could masquerade as stable functional effects, the voxelwise analyses were co-varied for the probability of gray-matter at each voxel in the brain [14]. Results represent the relation between anxious temperament and brain metabolism that cannot be explained by gross anatomical differences or registration error. In the NEC condition, significant (p<.05 two-tailed, multi-FDR corrected) correlations between anxious temperament and brain activity were detected in the left and right amygdala, left hippocampus, and the left brain stem (pontine nuclei region) (Fig. 3, pink; Table 1) [15]. Analyses of the brain activity during the ALN condition revealed that anxious temperament was significantly (p<.05 two-tailed, multi-FDR corrected) related to activity in the right amygdala, and right hippocampus (Fig. 3, blue; Table 1). Although some regions only reached statistical significance in one hemisphere, tests of hemispheric asymmetry revealed no significant differences (t's(32)<1.719, p's>.095), suggesting that hemispheric differences were an artifact of statistical thresholding.

**Table 1:** Brain Areas During Stressful Contexts Predict Anxious Temperament

Condition	Cluster				Local Maxima			*Location relative to anterior commisure (in mm)*		
	+/−	Area	Volume (in mm3)	Cluster Hemisphere	Area	Peak Hemisphere	Max t-value	x	y	z
**ALN**	+	Amygdala/ Hippocampus	64.45	R	Amygdala/ Hippocampus	R	5.6	7.53	−3.15	−9.35
*(p<.05, two-tailed, multi-FDR corrected)*	+	Hippocampus	24.41	R	Hippocampus	R	4.68	14.43	−12.45	−9.35
	+	Anterior Temporal Pole*	18.55	R	Anterior Temporal Pole*	R	4.89	16.23	8.75	−8.15
	+	Bed Nucleus of Stria Terminalis Region	7.81	R	Bed Nucleus of Stria Terminalis	R	4.46	5.03	0.65	−0.65
**NEC**	+	Amygdala/Anterior Temporal Pole*/ Hippocampus	364.26	R	Anterior Temporal Pole*	R	7.69	14.43	7.55	−8.15
*(p<.05, two-tailed, multi-FDR corrected)*	+				Amygdala	R	4.64	7.53	−0.65	−9.35
	+	Amygdala/ Anterior Temporal Pole*/ Hippocampus	314.94	L	Anterior Temporal Pole*	L	5.64	−17.48	9.35	−11.85
					Amygdala/ Hippocampus	L	5.32	−8.08	−4.35	−7.45
					Amygdala	L	4.83	−7.48	−1.85	−9.35
	+	Anterior Hypothalamus	17.82	L/R	Anterior Hypothalamus	R	5.12	1.23	1.85	−3.75
	+	Pontine Nuclei	16.60	L	Pontine Nuclei	L	4.71	−6.88	−14.95	−12.45
	+	Hippocampus	9.52	L	Hippocampus	L	4.31	−14.98	−11.85	−9.35
	+	Pontine Nuclei	8.30	R	Pontine Nuclei	R	4.76	8.73	−17.45	−15.65
**ALN and NEC**	+	Anterior Temporal Pole*	18.55	R	Anterior Temporal Pole*	R	4.89	16.23	8.75	−8.15
*(p<.05, two-tailed, multi-FDR corrected)*	+	Amygdala	11.72	R	Amygdala	R	4.62	7.53	−1.25	−9.35

Regions where anxious temperament was significantly (p<.05, two-tailed multi-FDR corrected) correlated with regional brain metabolism in the Alone (ALN) and No-Eye-Contact (NEC) conditions separately, and in both the ALN and NEC conditions combined with a logical AND conjunction analysis (ALN and NEC). Regions are presented with the direction of the correlation, brain regions involved, volume and hemisphere of cluster. We also report the local maxima for each anatomical region within the statistical cluster with its corresponding t-value and location (in millimeters relative to the anterior commisure). ^*^ Although the Anterior Temporal Pole may be important for the understanding of anxious temperament, we do not interpret these findings because there are highly metabolic muscles that border this portion of the brain that may influence the measures of brain metabolism. Therefore, in accordance with Drevets et al.,[34], we report but refrain from interpreting this finding.

We next used a logical AND conjunction analysis to identify the brain regions that across the different stressful contexts predicted individual differences in anxious temperament. Here we assessed the overlap between the brain regions that were correlated with anxious temperament in NEC with those that were correlated with anxious temperament in ALN. Analysis of the correlations in the NEC and ALN conditions revealed that the same regions of the right amygdala were significantly correlated with anxious temperament (p<.05, two-tailed multi-FDR corrected in both NEC and ALN) (Fig. 3, purple; Table 1) [16]. Thus, activity in a region of the right amygdala assessed in 2 different stressful contexts is consistently related to behavioral and physiological measures of stress responsivity assessed in those contexts. Notably, hierarchical linear regressions revealed the composite measure of anxious temperament to explain significant variance in amygdala activation beyond any one individual measure of stress (R^2^-Change's>.191, F-Change's(1,32)>10.742, p's<.003 Furthermore, there was only one voxel within the amygdala (as defined by our whole-amygdala ROI) that showed a significantly greater correlation with an individual measure of stress (cooing) than the composite measure of anxious temperament (R2-Chage = .081, F-Change(1,32) = 4.739, p = .031; other p's >.05). We also found that individual differences in metabolic activity in this overlapping amygdala region assessed during NEC or ALN were positively correlated with individual differences in the screening measures of anxious temperament that were determined 4.3 months prior to the FDG studies (early temperament vs. later NEC amygdala activity, r(33) = .335, p = .049; early temperament vs. later ALN amygdala activity, r(33) = .372, p = .028). This result suggests that the relation between anxious temperament and amygdala activation reflects the stable components of anxious temperament.

**Figure 4:**
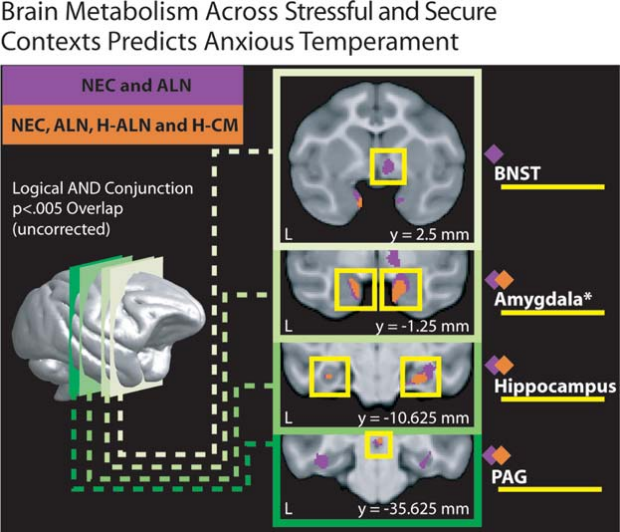
Regions where anxious temperament (Time 2) was significantly (p<.005, two-tailed uncorrected, two-condition logical AND conjunction) related to metabolism in both of the two stressful conditions [Alone (ALN) and No Eye Contact (NEC)] are shown overlaid on coronal slices of the study-specific MRI template in purple. Regions where anxious temperament (Time 2) was significantly (p<.005, two-tailed uncorrected, four-condition logical AND conjunction) related with brain metabolism in the stressful conditions as well as while animals were in the security of their home-cage [Home Alone (H-ALN) and Home with their Cage Mate (H-CM)] are shown in orange. Coordinates are in millimeters relative to the anterior commisure.

Although we predicted that amygdala activation is fundamental to anxious temperament, we were also interested in identifying the brain systems that interact with the amygdala to produce the behavioral and physiological changes. We further investigated additional regions that consistently demonstrated significant (p<.005, two-tailed, uncorrected) correlations between brain activity and anxious temperament in both the NEC and ALN conditions using a logical AND conjunction analysis [17]. This analysis revealed overlapping regions in bilateral amygdala, bilateral Bed Nucleus of Stria Terminalis (BNST), bilateral hippocampus, and periaqueductal gray (PAG) to be significantly related to anxious temperament (Fig. 4, purple; Table S1). Follow-up paired t-tests comparing the stressful (NEC and ALN) conditions to the home-cage conditions (H-ALN and H-CM) revealed that each of these regions, including the amygdala, had significantly greater activity during the stressful conditions (t's(34)>2.223, p's<.033). These results suggest that anxious temperament involves a neural circuit that is consistent with that previously characterized in preclinical mechanistic studies of stress and anxiety [18], [19].

Because we were interested in the stable neural circuitry of anxious temperament that we hypothesized would persist in non stressful environments, we examined whether individuals with anxious temperament would still display increased amygdala activation when assessed in the security of their home cage. Thus, the relation between anxious temperament and brain activation was assessed while the monkeys were in their home cages with their cage mates (H-CM) and when in their home cages without their cage mates (H-ALN). The H-ALN condition was studied to assess brain activity in a familiar environment in the absence of social interaction. Overall, activity from the overlapping amygdala region defined from the NEC and ALN conditions (Fig. 3, purple) was significantly less in the home cage conditions compared to the stressful conditions (t(34) = 2.626, p's <.013). To investigate the stability of the relationship between brain activation and anxious temperament across both stressful and secure contexts, we searched for regions where anxious temperament was significantly correlated with brain metabolism in each of the four conditions (NEC, ALN, H-CM, H-ALN). This logical AND conjunction analysis revealed that anxious temperament was significantly (p<.005 two-tailed, uncorrected) associated with activity in bilateral amygdala, bilateral hippocampus, and PAG in all four conditions (Fig. 4, orange; Table 2). Because cortisol samples were not always taken at the same time of day, we verified these results while statistically controlling for the time of the cortisol sampling. Controlling for time of day did not affect the highly significant (r's(31)>.493, p's<.004) relationships between anxious temperament and brain activity within the amygdala and the identified stress network. Importantly, the entire overlapping amygdala region identified from the NEC and ALN conditions (Fig. 1, purple) was significantly (p<.05 two-tailed, small-volume FDR-corrected within the amygdala) related to anxious temperament in the H-CM and H-ALN conditions. Moreover, the correlation between anxious temperament and this overlapping amygdala region was not significantly higher in the stressful conditions when compared to the secure conditions (t's(32)<1.313, p's>.19). We used an additional approach to further examine the relationship between anxious temperament and context dependent amygdala metabolism. Specifically, we correlated our measure of anxious temperament with the change in brain metabolism between each pair of conditions, as well as with the variability across all conditions within the amygdala and the identified stress network. These results revealed no significant correlations between anxious temperament and either the change between conditions (|r|'s(33)<.175, p's>.316) or the variability across conditions (|r|'s(33)<.179, p's>.304) within any of the regions tested. These results suggest our measure of anxious temperament in relation to individual differences in amygdala activity is not state related, but is stable across contexts. Overall, these results demonstrate that the relationship between anxious temperament and the amygdala, along with an extended stress network, is not restricted to the NEC and ALN conditions but extends to secure, non threatening, settings.

**Table 2:** Brain Areas Across Stressful and Secure Contexts Predict Anxious Temperament

Condition	Cluster				Local Maxima			*Location relative to anterior commisure (in mm)*		
	+/−	Area	Volume (in mm)	Cluster Hemisphere	Area	Peak Hemisphere	Max t-value	x	y	z
**ALN, NEC, H-ALN, and H-CM**	+	Amygdala	95.70	R	Amygdala	R	3.76	7.53	−2.45	−9.35
*(p<.005, two-tailed uncorrected)*	+	Amygdala	82.03	L	Amygdala	L	3.85	−8.08	−3.15	−9.35
	+	Anterior Temporal Pole*	80.32	R	Anterior Temporal Pole*	R	4.34	16.93	8.75	−8.15
	+	Anterior Temporal Pole*	59.08	L	Anterior Temporal Pole*	L	3.95	−11.88	6.85	−8.75
	+	Hippocampus	51.51	R	Hippocampus	R	3.63	13.13	−11.25	−9.95
	+	Hippocampus	8.30	L	Hippocampus	L	3.2	−15.58	−10.65	−9.35
	+	Genu of Corpus Collosum (white matter)	7.81	L/R	Genu of Corpus Collosum (white matter)	R	3.22	0.03	10.65	5.65
	+	Periaquaductal Gray	2.93	L/R	Periaquaductal Gray	R	3.19	0.63	−14.95	−1.25
	−	Visual Cortex	37.11	L	Visual Area V3	L	−3.51	−16.25	−31.25	8.125

Regions where conjunction analyses revealed anxious temperament to be significantly (p<.005, two-tailed uncorrected logical AND conjunction) correlated with regional brain metabolism in the stressful [Alone (ALN) and No-Eye-Contact (NEC)] and the non-stressful [Home Alone (H-ALN) and Home with Cage-Mate (H-CM)] conditions. Regions are presented with the direction of the correlation, brain regions involved, volume and hemisphere of cluster. We also report the local maxima for each anatomical region within the statistical cluster with its corresponding t-value and location (in millimeters relative to the anterior commisure). ^*^ See Caption for Table 1.

## Discussion

The current study defines a region of the amygdala that is consistently related to anxious temperament in young developing primates. Individual differences in activity of this amygdala region, assessed in four different contexts, predicted an individual's dispositional tendency to have anxious temperament. Consistent with the nature of temperament, longitudinal assessments of stress-related behavior and physiology revealed that the individual differences in stress reactivity were stable over time [13]. Along with the amygdala, the findings demonstrate an association between anxious temperament and an extended neural network previously associated with stress and emotion processing [18], [19]. Specific regions that are involved include hippocampus, a region of the basal forebrain that contains the BNST, and a region of brain stem that encompasses the PAG [20]–[23]. Importantly, all of these regions are highly interconnected with the amygdala, and likely work together to mediate stress-related behavior and physiological responses. The hippocampus is involved in memory and hypothalamic-pituitary regulation [21], [22], the BNST, considered part of the extended amygdala, mediates anxiety and is involved in autonomic and HPA regulation [20], [21], and the PAG mediates defensive behaviors including BI or freezing [23].

Although it has been suggested that increased childhood amygdala activity underlies the anxious disposition that increases children's risk to develop anxiety and depression, this is the first study to demonstrate this relationship using concomitant behavioral, physiological and functional brain measures assessed early in the life of a primate. This study shows that individuals with anxious temperament have increased amygdala reactivity across contexts varying in their degree of security and stressfulness and that it is not just amygdala activity that is elevated but also activity in an extended neural network downstream of the amygdala. The extended circuit uncovered in this study likely mediates the specific behavioral, emotional, pituitary-adrenal, and autonomic responses reported in children with anxious temperament. It is important to emphasize that the relation between individual differences in anxious temperament and brain activity was maintained even when brain activity was assessed in the security of an individual's highly familiar home environment. This finding suggests that individuals with anxious temperament have heightened activity in stress-related brain systems in a secure and therefore inappropriate context. Unlike other research on BI that has measured the phenotype in novel and unfamiliar contexts where it is normative to display anxiety, our findings suggest that the activation of neural activity in a stress-relevant circuit when in a safe and familiar context may be particularly significant in determining vulnerability to psychopathology in affected individuals. The readiness of these brain systems to respond could mediate the difficulty these individuals have in being able to relax in environments that others perceive as non stressful. Because increased amygdala reactivity is associated with a variety of conditions, it is possible that individuals with trait-like increased amygdala activity may be prone to develop various disorders depending on the influence of environmental and genetic factors. For these reasons, researchers should be careful not to interchange increased amygdala activity with “anxiety” or “anxious temperament”. Related to this, it is important to note that “anxiety” or “anxious temperament” are complex multi-dimensional constructs [24]. It is possible that the relationship between amygdala activation and anxious temperament reported here is unique to the specific conditions that were tested. However, we believe this to be unlikely since monkeys were tested in 4 different paradigms that differed considerably in context, degree of stressfulness, and adaptive response elicited. Despite these caveats, we believe that the findings presented here provide compelling evidence that trait-like over-activity of the amygdala and its accompanying neural circuit underlies the neurobiological substrate for the temperamental risk to develop anxiety and depression.

## Methods

### Subjects

One hundred and sixteen rhesus monkeys (***Macaca mulatta***) underwent behavioral testing (Screening, Time 1), and 36 (26 females) monkeys were selected to undergo a brain imaging experiment (FDG-PET, Time 2). Of these animals, 23 animals were selected to undergo another behavioral test approximately 1.5 years later (Time 3: Follow-up). All animals were pair-housed at the Harlow Primate Laboratory and the Wisconsin National Primate Research Center. These subjects were also part of a study that focused on genotype and brain function (Molecular Psychiatry, In Press). The average age was 2.7 (+/− .09 SEM) at the time of FDG-PET. Animal housing and experimental procedures were in accordance with institutional guidelines.

### FDG-PET Testing Paradigm

Each monkey was injected with FDG immediately preceding two stressful and two secure conditions. During exposure to the stressful conditions, FDG-uptake occurred and behaviors were monitored non-invasively using a video-recorder. Following 30-minutes of exposure to the experimental conditions, animals were anesthetized and cortisol samples were taken. The first stressful condition consisted of a separation in which the animal was relocated to a test-cage and remained alone for 30-minutes (ALN). The second stressful condition was the no eye contact (NEC) condition of the extended human intruder paradigm [6], [25], in which the animals are placed in the test-cage and a human intruder enters the room and stood still at a distance of 2.5 meters presenting their profile to the animal for 10 minutes. After the initial 10 minutes of NEC, the intruder left the room for five minutes, re-entered for five additional minutes of NEC, left the room again for five minutes, and re-entered for the last five minutes of NEC. This prevented habituation to the NEC condition over the 30 minutes of behavior testing. For cortisol measurements, plasma samples were taken approximately 4 minutes after behavioral testing (median: 4 min; range: 2–9 min). To measure FDG uptake during secure contexts, we assessed animals in their home-cage. This prevented observation of behaviors during the secure conditions. Each monkey underwent two 30-minute secure-condition scans in their home-cage, one in which the animals' cage-mate was removed and they were alone (H-ALN), and another when their cage-mate was present in the cage (H-CM). Scans we performed at least 1 week apart, and the order of conditions was counterbalanced.

### Behavioral Assessment

During each exposure to the NEC and ALN conditions behavior was assessed using standard methods by trained raters using a closed circuit television system [6]. Freezing was defined as a period of at least three seconds characterized by tense body posture, no vocalizations and no movement other than slow movements of the head. The frequency of coo vocalizations, were also assessed and are defined as being made by rounding and pursing the lips with an increase then decrease in frequency and intensity. To ensure that behavioral data were normally distributed, the duration of freezing behavior was log-transformed and frequency of coo vocalizations was square-root transformed.

### Cortisol

We assessed individual differences in plasma cortisol following stress exposure. Plasma was immediately separated from whole blood by centrifugation at 4°C and frozen at −70°C until assayed. Cortisol was measured in plasma samples using an enzyme immunoassay kit (Diagnostic Systems Laboratories, Webster, TX). The intraassay Coefficient of Variation (CV)% is 5.0% and interassay CV% is 8.0%. The detection limit for this assay is 0.5 µg/dL.

### MRI

MRI data were collected using a GE Signa 3T scanner (General Electric Medical Systems, Milwaukee, WI) with a standard quadrature birdcage headcoil using an axial 3D T1-weighted inversion prepared fast gradient echo sequence (TR = 8.648 ms, TE 1.888 ms, FOV = 140 cm, flip angle = 10°, NEX = 2, matrix = 512×512, voxel size = 0.2734 mm, 248 slices, slice thickness = 1 mm, slice gap = −.05 mm, prep time = 600, bandwidth = 15.63, freq = 256, phase = 224). Before undergoing MRI acquisition, the monkeys were anesthetized with ketamine (15 mg/kg) intramuscularly.

### FDG-PET Aquisition

Animals received intravenous (IV) injections into the saphenous vein of 10 mCi [18F]-flouro-2-deoxyglucose (FDG) (approximately .98 ml; median: .08 ml; range: .03 ml–2.4 ml) immediately before exposure to each of the 30-minute behavioral paradigms, during which FDG-uptake occurred. To minimize the effects of handling and injection, animals were adapted to all procedures associated with the handling and injection (with the exception of inserting the syringe) 5-days a week for up to 21 days prior to scanning. After the behavioral paradigm cotisol samples were taken and animals were anesthetized with 15 mg/kg of ketamine intramuscularly. Approximately 30 minutes after the cortisol sampling (median: 28; range: 20–57 min), subjects were fitted with an endotracheal tube and positioned in a sterotaxic head holder, and given isoflurane gas anesthesia (1–2%) for the duration of the 60-minute scanning procedure, during which integrated FDG-uptake from the behavioral paradigm was measured. Scanning was performed using the microPET P4 scanner (Concorde Microsystems, Inc., Knoxville, TN), which has an approximate resolution of 2 mm^3^ [26]–[28]. FDG-PET images were collected with a transmission scan, and reconstructed using filtered backprojection with attenuation and scatter correction.

### Pre-Processing

Each subject's anatomical image was transformed to the standard space of Paxinos, Huang & Toga [29] after the creation of a study-specific template. First each subject's T1-MRI image was manually stripped of non-brain tissue (extraction was performed by ASF using SPAMALIZE, http://brainimaging.waisman.wisc.edu/~oakes/spam/spam_frames.htm). Brain extracted MRI images were registered to a 6-brain template (c.f. [10], [25]) in the standard space, using a 9-parameter linear transformation using FMRIB Software Library's “flirt” tool (FSL; http://www.fmrib.ox.ac.uk/fsl/) [30]. Images were manually verified, and averaged to create a study-specific template in standard space. The brain-extracted MRI images in original space were then transformed to match this study-specific template using both linear 12-parameter affine, and 5^th^ order non-linear transformation using Automated Image Registration (AIR; http://bishopw.loni.ucla.edu/air5/) [31]. Images in standard space were segmented into the probability of gray-matter, white-matter and CSF using FSL-fast [32]. In addition to a study-specific template, this procedure resulted in both transformations from each subject's original T1-MRI to standard space, and a subject specific probability map, in which each point represented the probability of gray-matter at each voxel in standard space. Gray-matter probability maps were smoothed using a 4 mm Full Width Half Max (FWHM) Gaussian smoothing kernel to facilitate across subject statistical comparisons.

FDG-PET images were transformed into standard space based on the transformations derived from their anatomical data. In order to align each subject's FDG-PET to their T1-MRI image, we first created an FDG-PET template for each subject. Each subject's FDG-PET images was registered using a 6-parameter rigid body transform to the match the first FDG-PET image collected for that subject, using FSL [30]. Resulting images were averaged to create subject-specific FDG-PET templates. Original FDG-PET scans were then re-registered to match the subject-specific template using a 6-parameter rigid-body transformation with AIR [31]. These images were in-turn averaged, and the average image was transformed to match the subject's original space T1-MRI using a 6-parameter rigid-body transformation. The transformations to the FDG-PET-template were combined with the transformation to the original-space T1-MRI and the study-specific template. These transformations were then applied to the original FDG-PET images, and produced FDG-PET images in standard space. Standard-space FDG-PET images were scaled to correct for global intensity differences based on the mean FDG concentration across the whole brain. Globally scaled images were then smoothed using a 4 mm FWHM Gaussian smoothing kernel. All images were visually inspected to ensure accurate pre-processing before statistical analyses were performed.

### Statistical Analyses

Across subject statistical analyses were performed using an adapted version of Fmristat [33], [34] (http://www.math.mcgill.ca/keith/fmristat/; http://brainimaging.waisman.wisc.edu/fox/multistatic/). All FDG-PET analyses were performed across the whole-brain while controlling for both age and the probability of gray-matter on a voxelwise basis, using a multiple regression framework [14]. Conjunction analyses were performed across conditions using a logical AND conjunction analysis and a minimum statistic [16]. This test was chosen because it remains a valid test under violations of independence assumptions. This test allowed us to combine statistical parametric maps and identify regions of the brain that reach statistical significance in all of the individual test that compose the conjunction analysis. This test is equivalent to finding the intersection of voxelwise significance maps. Correction for multiple comparisons was performed using the multFDR threshold program written by Tom Nichols (http://www.sph.umich.edu/nichols/FDR/). This correction allowed us to correct for the False Detection Rate (FDR), or number of false positives, across multiple brain scans from the ALN and NEC conditions. Results from this test are reported as “p<.05, multi-FDR two-tailed corrected,” and represent areas that survived the multiple brain FDR threshold computed based on the ALN and NEC conditions. In order to investigate areas that were significant across both stressful and secure conditions, we used an uncorrected threshold of p<.005 (reported as, “p<.005, two-tailed uncorrected”) and computed a logical AND conjunction of the significant regions [16]. This result revealed regions that were significantly (p<.005, two-tailed uncorrected) correlated with anxious temperament (Time 2) when the monkey was exposed to both stressful and secure experimental conditions. Under assumptions of independence this would reflect a p-value of our reported threshold to the power of the number of conjunction tests, i.e. p<.005ˆ4 or .000000000625.

Follow-up analyses were performed by extracting the mean values of clusters of interest, and residualizing them for both the mean probability of gray-matter and age. Because the units of the residualized variables became meaningless, we also z-scored these variables before further analyses. Therefore, all follow-up analyses were performed on z-scored residualzed mean FDG values. Results are reported for correlations between the variables of interest; the difference between correlations, performed using a r to z transform, and comparing z-scores; as well as hierarchical linear regressions, in which we examine the unique variance accounted for by the variables of interest using the R^2^-Change statistic.

## Supporting Information

Table S1 Brain Areas Across Stressful Contexts Predict Anxious Temperament at Reduced Thresholds.
(0.10 MB DOC)
