# Supplementary material for: Trait-Like Brain Activity during Adolescence Predicts Anxious Temperament in Primates
Source: PLoS One. 2008 Jul 2;3(7):e2570. doi: 10.1371/journal.pone.0002570 (PMC2430534; doi:10.1371/journal.pone.0002570)
Supplement: Table S1 — Brain Areas Across Stressful Contexts Predict Anxious Temperament at Reduced Thresholds. (0.10 MB DOC) [file pone.0002570.s001.doc]

**Supporting Information for “Trait-like Brain Activity during Adolescence Predicts Anxious Temperament in Primates.”**

Fox, Andrew S.^1,3^, Shelton, Steven E.^2^, Oakes, Terrence R.^3^, Davidson, Richard J.^1,2,3^, Kalin, Ned H.^1,2,3^

*Departments of Psychology^1^, Psychiatry^2^ and the Waisman Laboratory for Brain Imaging and Behavior^3^ at the University of Wisconsin-Madison, Madison, WI 53706.*

**Materials include:**

Supporting Table 1

| **Table S1** |  | Brain Areas Across Stressful Contexts Predict Anxious Temperament at Reduced Thresholds | | | | | | | | | |
| --- | --- | --- | --- | --- | --- | --- | --- | --- | --- | --- | --- |
|  |  |  |  | |  |  |  |  |  |  |  |
| **Condition** | **Cluster** | | | | | **Local Maxima** | | | ***Location relative to anterior commisure (in mm)*** | | |
|  | **+/-** | **Area** | | **Volume (in mm)** | **Cluster Hemisphere** | **Area** | **Peak Hemisphere** | **Max t-value** | **x** | **y** | **z** |
| **ALN and NEC** | + | Amygdala/ Hippocampus | | 510.25 | R | Amygdala | R | 4.62 | 7.53 | -1.25 | -9.35 |
| *(p<.005, two-tailed uncorrected)* |  |  | |  |  | Hippocampus | R | 3.88 | 12.53 | -10.65 | -10.65 |
|  | + | Amygdala/ Anterior Temporal Pole*/ Hippocampus | | 249.02 | L | Anterior Temporal Pole* | L | 3.95 | -11.23 | 6.25 | -8.75 |
|  |  |  | |  |  | Amygdala/ Hippocampus | L | 3.85 | -8.08 | -3.15 | -9.35 |
|  |  |  | |  |  | Amygdala | L | 3.57 | -6.88 | -0.65 | -9.95 |
|  | + | Anterior Temporal Pole* | | 181.40 | R | Anterior Temporal Pole* | R | 4.89 | 16.23 | 8.75 | -8.15 |
|  | + | Hippocampus | | 117.68 | L | Hippocampus | L | 3.81 | 12.53 | -11.25 | -9.95 |
|  | + | Bed Nucleus of Stria Terminalis Region | | 109.13 | R | Bed Nucleus of Stria Terminalis | R | 3.92 | 5.03 | 0.05 | 0.65 |
|  | + | Genu of Corpus Collosum  (white matter) | | 19.78 | L/R | Genu of Corpus Collosum  (white matter) | R | 3.84 | 0.03 | 10.65 | 5.65 |
|  | + | Edge of Lateral Ventricle  (white matter) | | 12.45 | R | Edge of Lateral Ventricle  (white matter) | R | 3.55 | 8.73 | -21.25 | 4.35 |
|  | + | Periaquaductal Gray | | 12.45 | L/R | Periaquaductal Gray | R | 3.26 | 0.63 | -14.35 | -1.85 |
|  | - | Visual/ Parietal Cortices | | 1445.10 | L | Visual Area V2 | L | 4.28 | -4.38 | -32.45 | 5.05 |
|  |  |  | |  |  | Parietal Area PGM | L | 4.18 | -1.88 | -23.75 | 15.65 |
|  |  |  | |  |  | MST | L | 3.96 | -9.98 | -24.35 | 15.05 |
|  |  |  | |  |  | Visual Area V1 | L | 3.94 | -9.98 | -28.15 | 3.15 |
|  |  |  | |  |  | Visual Area V3 | L | 3.9 | -6.28 | -28.15 | 5.65 |
|  |  |  | |  |  | Parietal Area PEa | L | 3.84 | -5.58 | -29.35 | 16.85 |
|  | - | Visual Cortex | | 356.20 | R | Visual Area V1 | R | 3.77 | 13.13 | -33.75 | 5.05 |
|  |  |  | |  |  | Visual Area V2 | R | 3.23 | 23.13 | -33.15 | 6.25 |
|  | - | Visual Cortex | | 67.10 | L | Visual Area V4 | L | 3.71 | -21.88 | -28.75 | 11.85 |
|  |  |  | |  |  | Visual Area V2 | L | 3.51 | -21.28 | -33.15 | 9.35 |
|  | - | Visual/Temporal Cortices | | 63.50 | R | Visual Area V4 | R | 3.51 | 21.93 | -25.65 | 8.75 |
|  |  |  | |  |  | Temporal Area TEO, Medial Part | R | 3.33 | 25.63 | -19.35 | 3.75 |
|  | - | Visual Cortex | | 15.60 | L | Visual Area V3 | L | 3.59 | -12.48 | -28.15 | -3.75 |
|  | - | Left DLPFC | | 14.90 | L | Area 46/9 | L | 3.69 | -13.78 | 17.55 | 8.75 |

***Supplementary Table 1 Caption***

Regions where conjunction analyses revealed anxious temperament to be significantly (p<.005, two-tailed uncorrected logical AND conjunction) correlated with regional brain metabolism in the stressful conditions [Alone (ALN) and No-Eye-Contact (NEC)]. This table represents the NEC & ALN parts of table 1 at a reduced statistical threshold, as seen in the purple parts of figure 4. Regions are presented with the direction of the correlation, brain regions involved, volume and hemisphere of cluster. We also report the local maxima for each anatomical region within the statistical cluster with its corresponding t-value and location (in millimeters relative to the anterior commisure). * See Caption for Table 1.
